# Urinary biomarkers associated with podocyte injury in lupus nephritis

**DOI:** 10.3389/fphar.2024.1324540

**Published:** 2024-01-19

**Authors:** Zhiying Guo, Qianyu Guo, Xiaochen Li, Xinnan Gao, Liyun Zhang, Ke Xu

**Affiliations:** ^1^ Third Hospital of Shanxi Medical University, Shanxi Bethune Hospital, Shanxi Academy of Medical Sciences, Tongji Shanxi Hospital, Taiyuan, China

**Keywords:** systemic lupus erythematosus, lupus nephritis, podocyte injury, urine, biomarkers

## Abstract

The most prevalent and devastating form of organ damage in systemic lupus erythematosus (SLE) is lupus nephritis (LN). LN is characterized by glomerular injury, inflammation, cell proliferation, and necrosis, leading to podocyte injury and tubular epithelial cell damage. Assays for urine biomarkers have demonstrated significant promise in the early detection of LN, evaluation of disease activity, and tracking of reaction to therapy. This is because they are non-invasive, allow for frequent monitoring and easy self-collection, transport and storage. Podocyte injury is believed to be a essential factor in LN. The extent and type of podocyte injury could be connected to the severity of proteinuria, making podocyte-derived cellular debris and injury-related urinary proteins potential markers for the diagnosis and monitoring of LN. This article focuses on studies examining urinary biomarkers associated with podocyte injury in LN, offering fresh perspectives on the application of biomarkers in the early detection and management of LN.

## 1 Introduction

### 1.1 Lupus nephritis (LN) and podocyte injury

Any organ in the body can be impacted by the chronic autoimmune disease SLE. (Stanley et al., 2020). Among the major organs affected by SLE, the kidney is particularly susceptible, with 30%–60% of SLE patients developing kidney involvement. (Davidson, 2016). Within 5 years of diagnosis, between 10 and 30 percent of these people develop end-stage renal disease. (Gasparotto et al., 2020; Mok et al., 2023). Additionally, in these patients, LN is substantially linked to higher rates of morbidity and death. (Mok et al., 2023). LN is the most common immune injury following SLE involvement of the kidney and is linked to significant renal lesions. It is mainly characterized by glomerular involvement, (Lichtnekert, Anders, and Lech, 2022), including glomerular injury, as well as inflammation, cell proliferation and necrosis, leading to podocyte injury and tubular epithelial cell damage. Complement activation-induced immune complex deposition in various glomerulus areas and subsequent innate and adaptive immune system component activation are pathophysiological mechanisms of LN. (Bhargava et al., 2021). These processes in the glomerulus can result in various clinical, biological, and histological changes in renal function. Given the elevated death and morbidity rates of LN, particularly in those suffering from combined end-stage renal failure, early diagnosis and supportive treatment are crucial for preserving renal function, reducing death and morbidity rates associated with chronic renal failure and kidney disease, and minimizing drug-related toxicity. (Moroni et al., 2018; Gamal et al., 2023).

**FIGURE 1:**
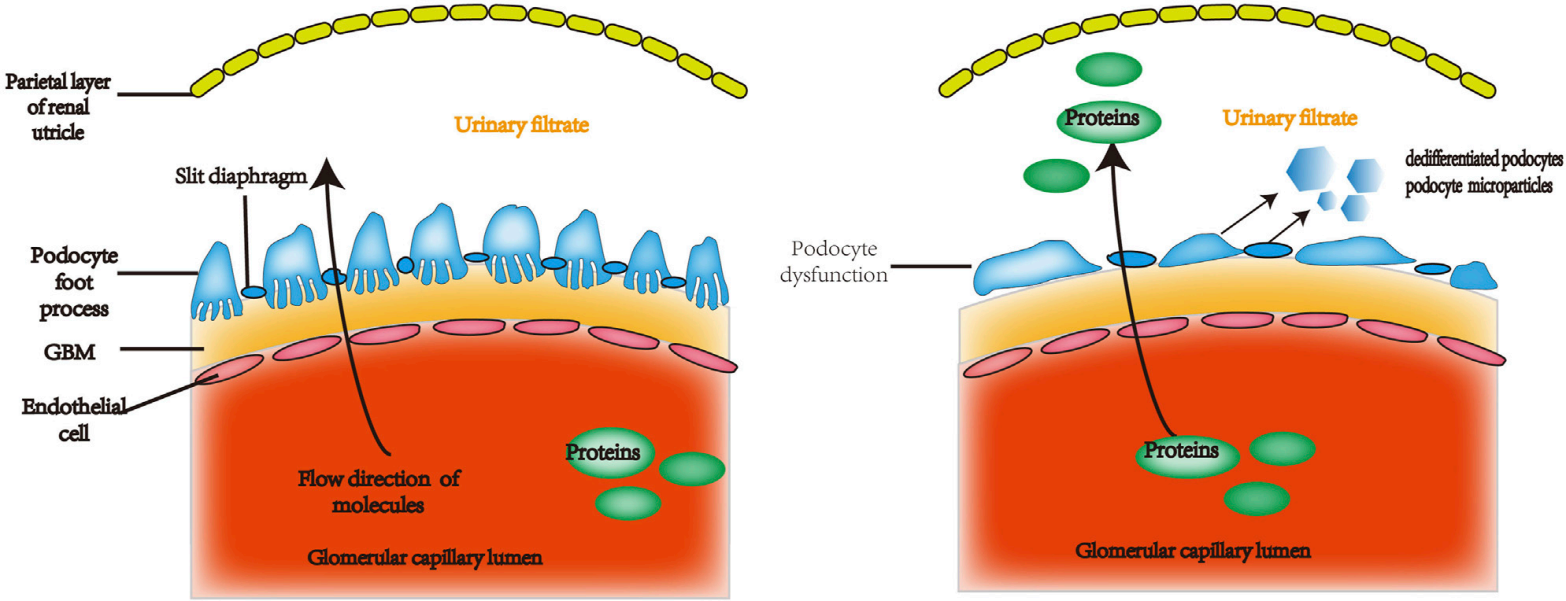
The glomerular filtration barrier is made up of perforated capillary endothelial cells, the GBM, and highly specialized, terminally differentiated cells called podocytes. Under normal circumstances, certain plasma constituents are filtered into the renal capsule lumen through the perforated endothelium, basement membrane, and podocyte fissure membrane. These membranes are incredibly permeable to water and small solutes, but essentially do not allow albumin or other proteins of equal or greater molecular weight to pass through. Proteinuria can be caused by defective podocyte shape and function, which can increase permeability to albumin and other proteins of the same size or greater. GBM: glomerular capillary basement membrane.

The podocyte is a highly specific, terminally differentiated cell with numerous foot processes that extend from the podocyte body and are linked by a slit diaphragm in the middle. A contractile system consisting of microfilaments made of actin, myosin, talin, vinculin, paxillin, and paladin mostly maintains the podocyte’s structure. (Wang et al., 2022a). Podocytes, endothelial cells, and the glomerular capillary basement membrane (GBM) make up the glomerular filtration barrier. (Li et al., 2007). Podocytes regulate glomerular filtration, contribute in local immunological and inflammatory responses, and preserve the glomerular vascular ring’s form. Additionally, Podocytes contribute to the GBM’s formation and recycling (Ishii et al., 2020) and the generation of paracrine substances that influence endothelial cell permeability and proliferation, such as vascular endothelial growth factor (VEGF). (Tufro and Veron, 2012). Podocyte malfunction is a major contributor to the development of proteinuria and is involved in the causes and progression of numerous renal disorders, including LN. (Sakhi et al., 2019) (Figure 1).

The significance of podocyte (visceral epithelial cell) damage in LN has been highlighted in numerous recent studies (Sakhi et al., 2019). Immune complex deposition in LN can directly or indirectly target podocytes, and podocyte dysfunction may contribute to the growth of glomerular lesions in LN. It has been postulated that podocyte damage happens early in the course of LN immune complex deposition and precedes irreversible glomerular damage. More than 30% of podocyte depletion leads to glomerular instability, ultimately resulting in glomerulosclerosis, and correlates with the severity of LN. (Bhargava et al., 2021). It seems that podocyte damage is a major factor in LN. It entails a number of pathways, such as interaction between immunological and parietal epithelial cells and disruption of the podocyte actin cytoskeleton. A recently discovered variant of LN called lupus podocytopathy can result from podocyte damage. (Sakhi et al., 2019). There is increasing interest in podocytopathy, which is defined on electron microscopy by the extensive loss of podocyte foot processes and is a particular histological feature in light microscopy. Therefore, It has been suggested that this is a different kind of LN. (Chen and Hu, 2018). The degree of proteinuria may be correlated with the type and extent of podocyte injury (structural or functional), and podocyte lesions may be a severe manifestation of podocyte change. (Yu et al., 2017). It has been shown that podocyturia is associated with the progression of LN and that podocytes can be found in the urine of LN patients, while healthy individuals or SLE patients with adequate kidney function do not have podocytes in their urine. Urinary podocyte count is associated with proteinuria and hematuria excretion scores. (Cui and Xie, 2017).

### 1.2 Biomarkers associated with podocyte damage in urine may together form a possible liquid biopsy modality for LN

Renal biopsy is considered the gold standard for the diagnosis, prognosis, and treatment of LN tissue. It enables the pathological groupings to be classified and the severity of renal involvement to be assessed based on active and chronic lesions. (Fanouriakis et al., 2020). However, it is an invasive and costly technique that is not suitable for tracking the effectiveness of treatment or detecting kidney pathology early on. The indications for repeat renal biopsy are controversial (Moroni, Depetri, and Ponticelli, 2016). Podocyte injury needs to be evaluated under an electron microscope, and the lack of electron microscopy in some medical institutions also makes it difficult to assess podocyte injury promptly. Currently, low plasma complement levels (mostly C3 and C4), anti-double-stranded DNA autoantibodies (anti-dsDNA), proteinuria, creatinine clearance, the urine protein/creatinine ratio, and other laboratory indicators of LN are used for monitoring LN activity in daily clinical routines. (Ligtenberg et al., 2022). Early detection and timely treatment can significantly impact the morbidity and mortality of LN. However, The diagnostic methods used now are not the best for early detection. (Caster and Powell, 2019; Akhgar et al., 2023). Therefore, further investigation into easily measurable biomarkers of LN with high predictive value is needed. (Stanley et al., 2020). Using non-invasive biomarkers to better monitor renal inflammation may aid in determining which patients are vulnerable for disease progression or therapy failure. (Radin et al., 2021).

Podocyte damage leads to molecular shedding from various podocyte sources into the urinary cavity, which can then be used as biomarkers of renal disease. In comparison to other sources of biological samples (such as tissue or serum), urine sampling is non-invasive, allows for regular monitoring, and enables self-collection, transportation, and storage. Additionally, urine biomarkers derived from urinary tissues, (Abedini et al., 2021), reflect the current diseased state and appear to be more helpful in investigating LN than serum markers. (Mok and Mohan, 2021). In conclusion, urine is a non-invasive biological sample that accumulates changes in the body’s biological systems and is not regulated by the body’s homeostatic mechanisms, allowing it to reflect earlier and more sensitive changes in the body caused by disease. The analysis of urine samples will contribute to a deeper understanding of biomarkers. Patients with active LN can be distinguished from those with inactive illness using a number of urine indicators, (Lindblom, Mohan, and Parodis, 2022), and various methods have been employed in cross-sectional studies to identify groups of biomarkers associated with LN. (Brunner et al., 2019; Tan et al., 2021).

Here, we present the main urine indicators linked to podocyte injury in LN that may serve as non-invasive biomarkers for the early diagnosis of LN and monitoring LN activity. We concentrated on indicators linked to podocyte injury because they may more accurately indicate renal inflammation and hence, LN activity.

## 2 Urinary biological markers related to podocyte injury

### 2.1 Urinary dedifferentiated podocytes

It has been studied how urinary podocyte shedding occurs in LN. Prior studies have demonstrated urinary podocytes predominantly survive but undergo dedifferentiation in patients with active LN, and compared to healthy controls, the percentage of apoptotic podocytes in urine is substantially lower. (Perez-Hernandez et al., 2016). According to this study, individuals with SLE, particularly those with active LN, had considerably higher urine levels of podocalyxin, podocin, synaptopodin, nephrin, and WT-1 (measured by protein blotting). These levels also showed a strong correlation with the degree of proteinuria and histological activity. (Perez-Hernandez et al., 2016). Protein levels of these dedifferentiated podocyte-associated molecules may serve as a noninvasive marker of glomerular disease progression in SLE patients. Associated proteins can be detected in SLE patients without the development of proteinuria, and theoretically the appearance of proteins from these dedifferentiated podocyte-associated molecules precedes the development of conventional proteinuria, but further experiments are needed to verify this.

Nephrin, podocin, and synaptopodin urine mRNA levels were discovered to be considerably greater in active LN patients compared to those with quiescent lupus, according to a later investigation. Urine podocin mRNA levels were a separate indicator of decreased renal function, whereas urine nephrin mRNA levels were associated with proteinuria and global disease activity but not to the histological category of LN. (Wang et al., 2007). The expression of podocyte related molecules in LN urine may reflect the activity of lupus. These markers can distinguish active lupus nephritis from inactive lupus nephritis.

### 2.2 Urinary podocyte microparticles

When cells activate and undergo apoptosis, phosphatidylserine (PS) externalizes from the plasma membrane, releasing a subtype of extracellular vesicles known as microparticles (MPs). (Martínez and Andriantsitohaina, 2017). In addition, it has been found that MPs of podocyte origin play an important role in the etiology of several glomerular and non-glomerular diseases and are a new early indicator of glomerular cell injury. (Burger et al., 2014). Extracellular vesicles (particles and exosomes) are recognized as biomarkers for many diseases such as lupus nephritis, diabetic nephropathy, preeclampsia, focal segmental glomerulosclerosis, and IgA nephropathy. (Farzamikia et al., 2021). To date, there have been no studies directly comparing the specificity of MP of podocyte origin in detecting these nephropathies.

A cross-sectional study showed a significant increase in MPs in urine samples from patients with SLE. Using flow cytometry, the urinary annexin V podocalyxin MPs of each individual were measured. In addition, anti-dsDNA antibody titers, proteinuria, erythrocyte sedimentation rates, and SLE Disease Activity Index (SLEDAI) scores were all positively connected with urinary podocyte-derived MP levels. Patients with SLE who had modest disease activity and those who had moderate or more disease activity might be distinguished from one another using podocyte-derived MP levels. More MPs generated from podocytes were excreted in the urine in active LN. Urinary podocyte-derived MP levels were higher in patients with LN than in those without LN, and multiple morphologic podocyte lesions were present in different pathological types of LN. The levels of urinary podocyte-derived particles were closely related to the activity index and ultrastructural changes of podocytes. Urinary podocyte-derived MPs were subjected to Receiver Operating Characteristic (ROC) curve analysis in order to distinguish SLE patients with active LN from those without LN (AUC: 0.962 (95% CI 0.905–1)). Urinary podocyte-derived MPs had an area under the ROC curve of 0.789 (95% CI 0.62–0.958) for the diagnosis of LN disease activity. (Lu et al., 2019).

According to these results, variations in urine podocyte-derived MP levels may be utilized to measure and track the activity of SLE disease and to distinguish between SLE patients who have active LN and those who do not. (Lu et al., 2019). Measurement of 24-h proteinuria in urine samples is a classic biomarker for the assessment of LN, which in one way or another reflects the final renal outcome. Proteinuria is partially consistent with changes in the activity of histologic markers of LN, with a significant increase in the level of MP of urinary podocyte origin observed with the severity of histologic features. Some animal experiments have shown the presence of podocyte-derived MP in the urine in the early stages of diabetic kidney injury, and podocyte MP was detected in the urine before proteinuria, (Sullivan et al., 2021),but there are no studies on whether or not podocyte-derived MP occurs before proteinuria in patients with LN. The small sample size of this study may have affected the reliability of the results, thus larger samples and studies with prospective follow-up are required. Second, this study was unable to link greater podocyte MP numbers to the progression of renal illness, implying that more research is needed. It is still necessary to investigate if MPs may be utilized as novel biomarkers for the early detection and tracking of disease activity in SLE and LN.

### 2.3 Urine soluble urokinase plasminogen activator receptor (suPAR)

The suPAR receptor is for fibrinogen-activating enzymes and converts fibrinogen to fibrinolytic enzymes. It is involved in a variety of biological activities, including chemotaxis, cell adhesion, endothelial cell function, and immunological modulation. (Thunø, Macho, and Eugen-Olsen, 2009).

In experimental models of focal segmental glomerulosclerosis (FSGS), circulating suPAR has been shown to activate podocyte β3 integrins in natural and transplanted kidneys, leading to loss of podocyte foot processes, proteinuria and FSGS-like glomerulopathy. SuPAR is not the direct source of podocyte injury *in vitro* or *in vivo*, according to recent experimental findings on human podocytes and two animal models. (Musiała et al., 2022). Nonetheless, by forming signaling complexes with other transmembrane proteins, such as activating the podocyte αvβ3 integrin, it does connect innate immune activity to the preservation of the slit septum. Activation of this receptor and its downstream pathway activates small guanosine triphosphatase, leading to the loss of podocyte foot processes, proteinuria, glomerular damage and loss of renal function. (Hladunewich et al., 2022). Prolonged exposure to elevated levels of suPAR directly affects the kidneys through pathological activation of αvβ3 integrins expressed in podocytes, resulting in proteinuria. (Hayek et al., 2020).

Increased suPAR concentrations may serve as a specific circulating risk factor for focal segmental glomerulosclerosis (FSGS). (Musiała et al., 2022). Elevated suPAR levels have been found in up to two-thirds of FSGS patients. (Musiała et al., 2022). however, further clinical studies have observed increased suPAR concentrations in other glomerular and proteinuric diseases, suggesting that plasma suPAR accumulation is not a specific biomarker for FSGS. Some studies have shown that SuPAR is a biomarker that can be used to stratify patients and determine which SLE patients are most likely to experience organ damage in the first 5 years of their illness. (Enocsson et al., 2020).

The levels of suPAR in the serum and urine were significantly higher in SLE patients than in healthy controls. In addition, levels were higher in LN patients than in non-LN patients. Moreover, suPAR had a stronger correlation with disease activity, and showed significantly higher expression in the kidney tissue of LN patients, correlating with the activity of pathological lesions. (Wen et al., 2018). Recent longitudinal cohort data studies show that urinary suPAR levels correlate with changes in LN activity, with a significant decrease in urinary suPAR levels as LN disease activity improves. (Burcsár et al., 2021). Urinary suPAR levels are a promising biomarker for non-invasively predicting LN activity.

### 2.4 T-cell immunoglobulins and mucins 1 (Tim-1)

Tim-1 is a crucial gene that regulates T helper cell development. (Kong et al., 2020). Tim-1 is expressed by CD4^+^ T cells, which facilitates T cell activation through co-stimulatory signals, starts transcription during the early stages of antigen stimulation, contributes to T cell proliferation and differentiation, and prevents the onset of peripheral tolerance. (Li et al., 2020; Xu et al., 2020; Zhou et al., 2020). These results indicate that Tim-1 is one of the more crucial genes that may regulate T cells and is probably an immunological marker.

It has been demonstrated that LN activates inflammatory responses, which in turn trigger autophagy. Tim-1 expression, autophagy, and inflammatory responses are elevated in LN mice. In an LN cell model, Tim-1 stimulates autophagy and reduces the inflammatory response. In the same cell model, Tim-1 promotes IgG-induced podocyte proliferation and inhibits apoptosis. Tim-1 also attenuates the inflammatory response in LN podocytes by inducing autophagy. Furthermore, Tim-1 significantly promotes IgG-induced podocyte proliferation by inhibiting apoptosis. In IgG-treated podocytes, the autophagy inhibitor counteracted Tim-1’s effects on inflammatory cytokines and autophagy-associated proteins. (Yu et al., 2021). In summary, it can be concluded that Tim-1 is a possible novel target for LN treatment since it mediates autophagy, protecting podocytes from LN-induced damage.

Because of its increased expression in the kidneys and urine during renal injury, TIM-1 is a type I transmembrane protein that was first known as the kidney injury molecule 1 (KIM-1). (Thomas et al., 2016; Nozaki et al., 2023).

One study enrolled 61 SLE patients and 69 healthy controls, and serum TIM-1 levels were measured by ELISA. The findings demonstrated that serum TIM-1 levels in SLE patients were considerably lower than in controls, and that there was not a significant distinction in serum TIM-1 levels between patients with and without LN. Additionally, Serum TIM-1 levels did not significantly correlate with the activity of SLE illness. (Yuan et al., 2016).

A total of 154 SLE patients (94% female) with active LN, 32 inactive LN, and 49 non-renal SLE were recruited from Shanghai Jiao Tong University’s Renji Hospital as part of a retrospective cohort research. The patients were all 18 years of age or older. Controls compromised of 55 age- and sex-matched healthy individuals. Levels of u-cystatin-C, u-MCP-1, u-KIM-1, and u-VDBP were considerably higher in patients with active LN than in those with SLE without renal involvement. Moreover, u-cystatin C, u-MCP-1, and u-KIM-1 levels were considerably higher in the active LN group than in the inactive LN group. The present investigation showed that KIM-1 more accurately represents renal disease when used in conjunction with other UBM applications. (Liu et al., 2020). Additionally, it has been demonstrated that KIM-1 plays a significant role in a variety of urine biomarker combinations, including those that are used to forecast alterations in renal disease. (Brunner et al., 2016; Gulati et al., 2017; Ding et al., 2018).

In a recent study, a prospective urine analysis of 10 protein markers standardized to urine creatinine, namely, ALCAM, cystatin-C, hemopexin, KIM-1, MCP-1, NGAL, PF-4, Timp-1, TWEAK, and VCAM-1 by ELISA, was conducted on 84 pediatric patients who met ≥4 ACR criteria for SLE. Patients with active LN had substantially higher urinary concentrations of ALCAM, KIM-1, PF4, and VCAM-1 than to those with active non-renal SLE, inactive SLE, and healthy controls. (Soliman et al., 2022). This suggests a correlation between KIM-1 and lupus nephritis (LN) activity.

According to Yuji Nozaki et al., proteinuria and uKIM-1 levels were higher in active LN than in inactive LN in patients with SLE, and both uKIM-1 and proteinuria reduced as treatment intensified. In renal disease, uKIM-1 levels were linked to the percentage of glomerular crescent formation. Furthermore, at 12 months after therapy, individuals who had increased baseline uKIM-1 levels had substantially more intense eGFR and reduced LN disease activity. According to these findings, there may be a relationship between uKIM-1 levels and renal histological abnormalities as well as LN disease activity, and they may also be a predictor of therapy response. (Nozaki et al., 2023).

### 2.5 Calcium/calmodulin-dependent protein kinase IV (CAMK4)

CAMK4, a CAMK family member, is a versatile serine/threonine kinase that controls multiple components of the immune response. (Koga and Kawakami, 2018). CAMK4 affects podocyte motility by activating GTPases Rac1 and RhoA and phosphorylating the scaffold protein 14-3-3β. This leads to the release and degradation of synaptopodin and is directly involved in multiple etiologies of podocyte injury. It is elevated in nonautoimmune podocyte lesions as well as autoimmune podocyte lesions in humans and mice. Additionally, through downregulation of nephrin and synaptopodin expression, and interfering with slit diaphragm function and cytoskeletal dynamics, (Maeda et al., 2018), targeted delivery of CAMK4 inhibitors preserved podocyte architecture, prevented the progression of glomerulonephritis in mice predisposed to lupus, and repaired mice’s podocyte damage brought on by adriamycin. (Tsokos and Tsokos, 2019).

IgG from LN patients upregulates CAMK4 expression in podocyte culture, and it has been shown that urinary podocytes from patients with active LN stain positive for CAMK4. In addition, in comparison to individuals who did not have renal involvement or who had clinical improvements following treatment, total urothelial CAMK4 mRNA expression was higher in active LN. CAMK4 mRNA levels were elevated only in urinary podocytes from patients with active LN, whereas urinary CAMK4 mRNA expression was minimal in patients with a clinical response. The finding that urinary podocyte CAMK4 mRNA can distinguish between active and inactive LN introduces a potentially new non-invasive method to observe the activity of LN disease. (Bhargava et al., 2021).

### 2.6 The ubiquitin carboxy-terminal hydrolase L1 (UCH-L1)

The UCHL1 gene, also known as neuron-specific protein gene product 9.5, is located on chromosome 4 (4p14) and encodes a peptide of 223 amino acids. (Yu et al., 2008). Liu et al. discovered UCH-L1 in human IgA nephropathy and LN specimens using a pre-embedding immunoelectron microscopy technique with gold and horseradish peroxidase labeling in the cytoplasm and podocyte protrusions in 2008.

It was found that UCH-L1 appears *ab initio* in LN and is linked to podocyte injury, but not in the glomerular podocytes of healthy kidneys. (Meyer-Schwesinger et al., 2009; Zhang et al., 2015).With the exception of UCH-L1, no particular marker proteins that were present in normal LN podocytes but lacking or elevated in sick podocytes were found. Thus, UCH-L1 may predict prognosis or the extent of podocyte injury and serve as an indicator of histological distinction between healthy and sick podocytes. (Meyer-Schwesinger et al., 2009).

The intensity of UCH-L1 in the kidney is correlated with metabolic activity and is broadly dispersed in the epithelial cells of the glomerulus, tubules, and collecting ducts. (Zhang et al., 2013). Recent research has shown that a number of glomerulonephritis types, including membranous nephritis, IgA nephropathy, and LN in kidney biopsies from different human nephritis cases, are linked to a significant elevation of UCH-L1 in podocytes. (Liu et al., 2009). Through its ability to cause structural disruptions in the cytoskeleton of podocytes, UCH-L1 could be a major player in the pathogenesis of glomerulonephritis. (Zhang et al., 2016).

UCH-L1 may not directly regulate RhoA/synaptopodin, but rather harm the podocyte cytoskeleton by regulating plakoglobin, which may be a viable target for kidney disease therapy later on. (Fang et al., 2020). By regulating the quantity of proteases, UCH-L1 controls the breakdown of proteins in the kidneys. When proteasome abundance is out of balance, renal cells, especially endothelial cells and podocytes, become more liable to damage. (Radón et al., 2018).

Renal biopsies samples from LN patients showed elevated and positively linked expressions of UCH-L1 and NF-κB. UCH-L1 expression is upregulated when NF-κB is activated, which is followed by changes involving additional podocyte components such nephrin and snail. (Zhang et al., 2013). Elevated NF-κB and UCH-L levels in human kidney biopsies positively linked with the frequency of sick podocytes in a number of cases of immune complex-mediated membranous glomerulonephritis, including LN. (Zhang et al., 2015).

UCH-L1 may predict the level of podocyte injury or the prognosis and serve as an indicator of histopathological distinction between podocytes in health and those in sickness. Additionally, UCH-L1, a significant NF-κB downstream target gene, might offer a targeted treatment for LN. However, high expression of UCH-L1 is seen in human β-cells and neurons, and cutting it down could make diabetes, Parkinson’s disease, or Alzheimer’s disease more common. Therefore, targeted reduction of UCH-L1 in the kidneys alone is important for patients with LN; moreover, care should be used when using UCH-L1 as a treatment drug for LN. (Cui and Xie, 2017). According to the current work, UCH-L1 expression is regulated by A20 via signaling pathway of NF-κB, and A20 deficiency could be a significant factor in the pathophysiology of lung disease. (Sun et al., 2020). Furthermore, there is currently no research on urinary UCH-L1 in LN patients, making it a potential candidate as a non-invasive urinary biomarker for this condition.

### 2.7 Metabolic fingerprints

Metabolic fingerprinting is closely related to genomics and proteomics because metabolites are the end-products of gene expression, metabolites at the end of the pathway can accurately indicate a patient’s status in real-time, and metabolic profiling can be easily constructed without the need for costly or cumbersome sequencing/immunoassays, (Nemet et al., 2020), and solves the problem of delayed diagnosis and high cost of current biomarkers for genomic and proteomic biomarkers, metabolic biomarkers provide a more distal characterization of the pathology and physiological processes, which are more sensitive to slight changes in the state of health. (Wang et al., 2022b).

A recent study constructed a discovery cohort of 731 individuals, including 357 SLE patients and 374 healthy controls (HC), and a validation cohort of 184 individuals (SLE/HC, 91/93). Each SMF was directly recorded by nano-assisted laser desorption/ionization mass spectrometry (LDI MS) using 1 μL of serum within 1 min Sparse learning of SMFs enabled SLE identification with a sensitivity/specificity and area under the curve (AUC) of up to 86.0%/92.0% and a discovery cohort of 0.950. The great degree of consistency revealed by sparse learning in the discovery and validation cohorts has demonstrated SMF’s superiority in the diagnosis of SLE. The investigation was based on an optimized diagnostic model that included four metabolite groups: imidazoleoleoleacetic acid, 2-hydroxyadipic acid, glucose, and pseudouridine. The group was validated further in a small sample of SLE *versus* RA patients. These four putative biomarkers did not correlate with SLEDAI, implying that they are targeted toward SLE diagnosis, however, the ability to assess biomarkers is limited. (Li et al., 2023).

Machine learning of serum metabolic fingerprinting (SMFs) was developed in another study to identify SLE activity in pregnant women. The hollow cobalt oxide/carbon (Co3O4/C)-composite assisted laser desorption/ionization mass spectrometry (LDI MS) platform was used to directly extract smf. The EN method was optimized to develop a diagnostic model that differentiated between active SLE, inactive SLE, and HC in pregnant women using metabolic fingerprints derived from approximately 0.1 L of serum in 1 s without enrichment. The mean AUC values for distinguishing active SLE from inactive SLE and healthy controls were 0.985 and 0.990, respectively. To simplify the direct investigation of SLE episodes, the study created a simpler metabolite panel (acetoacetic acid, glucose, alanine, α-ketoisovalerate). (Wang et al., 2022b).

According to one study, urine metabolic fingerprints (UMFs) may be extracted using polymer@Ag-assisted LDI-MS, and supervised machine learning techniques like sparse learning can be used to diagnose LN. We quickly and easily acquired LDI-MS metabolic fingerprints of natural pee using just 1 mL of urine, without the need for enrichment or purification, by optimizing polymer@Ag. This study suggests a new diagnostic paradigm to identify clinical kidney disease subtypes by fusing UMFs and urine protein levels (UPLs). For patients with active LN, the model is known as a two-step noninvasive diagnostic model. First, we separated individuals with renal illness from a control group with an AUC of 1.00 by using UPLs (>0.5 g/24 h), which is the gold standard for clinical diagnosis of renal disease. Instead of using UPLs with an AUC of 0.52 to distinguish patients with active LN kidney disease from those with active non-LN kidney disease, we employed UMFs with an AUC of 0.89 in the second phase. It is important to remember that the first and second phases’ AUCs of 1.00 and 0.89, respectively, depend on the sequential analysis method. (Yang et al., 2020).

## 3 Conclusions and perspectives

The study of urine as a non-invasive information source for people with LN is gaining popularity. In LN studies, urine biomarkers appear to have an advantage over serum markers because they can guide treatment selection by characterizing intrarenal biology, tracking treatment response longitudinally, and determining the type or activity of nephritis non-invasively. Furthermore, urine biomarkers can offer real-time information on biological pathways and include data on the entire kidney, not just the tissue sampled at biopsy. As a result, these features have the potential to replace renal biopsy as a liquid biopsy method. Currently, the study of urinary markers in lupus nephritis mainly focuses on a portion of immune-related cytokines and proteins. LN is a type of glomerulonephritis caused by immune complex deposition, and its main pathogenesis is that immune complexes are deposited to the tunica, subendothelium, or subepithelium causing inflammation, which leads to impaired renal filtration barrier and produces proteinuria. The filtration barrier consists of the vascular endothelium, the glomerular basement membrane, and the podocytes, which are the last layer of the filtration barrier. Internationally, LN is categorized into six types of LN by the deposition of immune complexes to different locations in the kidney, and each type of LN can cause damage to the podocytes. Biomarkers directly associated with podocyte cell injury may be more relevant to these specific pathologic processes. Podocyte-associated biomarkers have some advantages in kidney diseases such as lupus nephritis (LN) because they provide more direct information about the extent of podocyte injury and the functional status of the kidney. Despite these advantages of podocyte-associated biomarkers, more studies are still needed to validate their accuracy, reliability, and clinical utility in clinical applications. In addition, a combination of multiple biomarkers, as well as other clinical and laboratory parameters, may be the most effective way to evaluate patients with LN.

Numerous investigations have highlighted the significance of podocyte damage in LN, and certain urinary markers associated with podocyte injury have been examined in part, such as urinary depolarized podocytes, urinary podocyte particles, suPAR, TIM-1, and CAMK4. However, urinary UCH-L1 in patients with LN has not yet been intensively investigated (Table 1), and the existing studies in this area are still relatively limited, focusing mainly on a small number of markers. Current studies are able to measure many proteins in urine that are involved in LN pathophysiology, and patients with acute LN can be distinguished from those with quiet illness by a number of proteins in their urine. (Fawzy et al., 2022). However, there are few longitudinal investigations, and those that are have not yet shown biomarker combinations that are more accurate at forecasting LN outcomes than standard clinical criteria. (Hoover et al., 2020). and larger prospective studies are necessary to validate these findings. The relationship between podocyte biomarkers and kidney function impairment and LN disease activity has been the subject of numerous investigations. Urine biomarkers could contain certain proteins, cytokines, compounds, etc. Physicians may be able to gain a better understanding of a patient’s condition and make early modifications to treatment plans by noninvasively monitoring changes in these markers. Nevertheless, additional investigation and verification are still required to incorporate podocyte indicators into the clinical LN treatment process. It is also necessary to create and enhance guidelines for clinical use and standardized assessment techniques.

**TABLE 1:** Summary of urine markers associated with LN podocyte injury.

Urine biomarkers	Diagnostic value	Prognostic utility
urinary dedifferentiated podocytes	Urinary mRNA levels of nephrin, podocin and synaptopodin were significantly higher in patients with active lupus nephritis than in patients with quiescent lupus Perez-Hernandez et al. (2016)	Urinary nephrin mRNA levels are associated with proteinuria and systemic disease activity, and urinary podocin mRNA levels are an independent predictor of decreased renal function Wang et al. (2007)
urinary podocyte microparticles	Changes in urinary podocyte-derived MP levels can be used to differentiate SLE patients with active lupus nephritis Burger et al. (2014)	Can be used to assess and monitor SLE disease activity
Urine soluble urokinase plasminogen activator receptor	Serum and urine levels of suPAR were significantly higher in SLE patients than in healthy controls, and higher in LN patients than in non-LN patients Wen et al. (2018)	suPAR has a strong correlation with disease activity and correlates with the activity of pathological lesions Wen et al. (2018)
T-cell immunoglobulins and mucins 1	Serum TIM-1 levels were significantly lower in patients with SLE compared to healthy subjects Yuan et al. (2016) and urinary TIM-1 levels were significantly higher in active LN compared to SLE patients without kidney involvement Brunner et al. (2016)	uTIM-1 levels were significantly elevated in the active LN group, Brunner et al. (2016) uTIM-1 has been shown to be an important component of several different urinary biomarker combinations, including for predicting pathological changes in the kidney Brunner et al. (2016), Ding et al. (2018), Gulati et al. (2017)
Calcium/calmodulin kinase IV (CaMK4)	Elevated total urocyte CAMK4 mRNA expression in active LN compared to patients with clinical response after treatment and patients without renal involvement Bhargava et al. (2021)	Urinary podocyte CAMK4 mRNA can distinguish active and inactive LN Bhargava et al. (2021)
The ubiquitin carboxy-terminal hydrolase L1 (UCH-L1)	UCH-L1 is not expressed in glomerular podocytes of normal kidneys, but is expressed *de novo* in lupus nephritis and associated with podocyte injury, no lupus nephritis urine-related studies are available	

The dependability of podocyte-associated biomarkers is significantly impacted by sample variability and assay consistency. Different laboratories, different periods, or various methodologies may yield inconsistent results. The primary causes of sample variability are variations in the sources and methods of sampling. There may be variations in biological samples between patients due to individual, genetic, and environmental factors. These variations could cause patient-to-patient variability in biomarker levels. Variations may also be introduced by different sampling strategies. The way urine is collected and the conditions under which it is handled and preserved, for instance, may affect the assessment of foot cell biomarkers. The primary factors that define assay consistency include variations in batches, laboratories, etc. Results from multiple laboratories using various experimental techniques, tools, or reagents may not always agree. Such disparities can be minimized by using standardized laboratory procedures and technologies. There could be some variation across batches of reagents or equipment even within the same laboratory. Consistent measurements can be maintained with routine calibration and observation using quality control samples. We can create and implement standardized procedures for sample collection, processing, and measurement to overcome these problems and guarantee consistent outcomes over time and between laboratories. To maintain uniformity throughout the laboratory, quality control samples are used for routine laboratory calibration. To gain a better understanding of how sample variability and assay consistency affect outcomes, do research at several medical facilities. Assay consistency is mainly characterized by laboratory differences, batch differences, etc. Different laboratories may use different experimental methods, instruments or reagents, which may lead to inconsistent results. Standardized laboratory methods and processes can reduce such differences. Within the same laboratory, there may be some variability in different batches of reagents or instruments. Regular calibration and monitoring with quality control samples can help maintain consistent measurements.

Future studies should aim to identify additional biomarkers and improve their sensitivity and specificity in detecting podocyte injury and inflammation in LN. Advances in high-throughput sequencing and proteomics may help identify novel biomarkers with greater accuracy and specificity. (Aljaberi et al., 2019; Fava, Raychaudhuri, and Rao, 2021). In addition, a combination of multiple biomarkers may improve the accuracy of diagnosis and increase the predictive value of these tests. It would be valuable to examine the connection between the activity of diseases and urine biomarkers at different stages of LN. Further studies are required to find the best time and frequency of sampling for these biomarkers and their utility in predicting treatment response and guiding treatment decisions. With more studies focusing on podocyte injury, new non-invasive urine biomarkers are likely to emerge soon, providing new ideas for early diagnosis of LN, monitoring treatment response, and selection of treatment options, with a significant impact on the quality of life of patients with LN.
